# Design and application of polyurea microcapsules containing herbicide (oxyfluorfen)

**DOI:** 10.1080/15685551.2020.1816344

**Published:** 2020-09-08

**Authors:** Jayprakash Rao, Amar Nath Chandrani, Anil Powar, Sudeshna Chandra

**Affiliations:** a Research and Development Laboratory, Indofil Industries Limited, Thane, India; b Department of Chemistry, Sunandan Divatia School of Science, SVKM’s NMIMS (Deemed to Be) University, Mumbai, India

**Keywords:** Polyurea, microcapsules, core to shell ratio, interfacial polymerization, encapsulation efficiency

## Abstract

Polyurea, a controlled release material, has been widely applied in agricultural fields due to high thermal stability and low cost. In this article oxyfluorfen polyurea microcapsules suspension was successfully prepared by interfacial polymerization using diisocyanate and polyamines such as Ethylenediamine, Hexamethylenediamine, Diethylenetriamine in presence of green solvent, i.e., N,N-dimethyldecanamide. The microcapsule suspension of oxyfluorfen has not been researched yet by using solvent N,N-dimethyldecanamide and polyamines. The effect and the type of diamines on the morphology and properties of the microcapsules have been investigated. The synthesized microcapsules were characterized by scanning electron microscope, ultraviolet spectrometry, Fourier transform iInfrared spectrometer, thermogravimetric analysis and particle size analyser. The effect of the core to shell ratio on encapsulation efficiency and release kinetics were also studied. The oxyfluorfen microcapsules had an excellent encapsulation efficiency (98.2%) using EDA as the monomer and Release kinetics depended upon the type of monomers used and also on core to shell ratio used (6.5:1, 5:1, 4:1). As core to shell ratio was increased the encapsulation efficiency was found to decrease. Prepared Microcapsules when sprayed on paddy crop was found to be safe in comparison with Emulsifiable concentrate sample.

## Introduction

In agriculture, controlled release techniques are used to improve the efficiency of herbicides, insecticides, fertilizers, fungicides and bactericides [1]. Pesticides are important labour-savings agrochemicals, used for improving the quality and quantity of agricultural products. However, excess use and misuse of pesticides have resulted in environmental pollution having adverse effects on the ecosystem and loss of diversity [2] Controlled release techniques can improve the utilization of pesticides, as well as reduce the frequency of agrochemical application [3].

For many years, the main purpose of developing formulations was to facilitate the transport of active ingredient to its target where formulator’s concern was largely with the physico-chemical properties of the formulation in bulk and when dispersed in the carried medium usually water [4]. This could be addressed satisfactorily with a range of relative simple formulations, but with considerable limitations. For example, emulsifiable concentrates have been extensively used for the delivery of herbicides. The formulations are generally simple to make, have high biological activity and are chemically stable. However, issues related to phytotoxicity, acute operator toxicity and ‘green’ pressures to minimise the impact of solvents on the environment have driven the industry to develop alternative formulation [4].

Microencapsulation technology has received huge attention in the past few years for its use in many industries such as health, cosmetic, food, agricultural etc [5]. In recent years, microencapsulation of pesticides has been demonstrated to be promising alternative for resolving and addressing such issues [5,6]. Microencapsulation can be beneficial to the manufacturers for delivering a product with less toxic effect and having controlled release. At present, many methods have been developed for microencapsulation including interfacial polymerization [7,8], *in situ* polymerization [9,10], complex coagulation and suspension polymerization [11,12].

Microencapsulation can be done by interfacial polycondensation reaction in solvents such as p-xylene, benzene, toluene, and cyclohexane [13]. Microcapsules can also be obtained by solvent evaporation wherein the commonly used solvents are chloroform and o-xylene, which are harmful and dangerous [14]. Xylene has been main solvent for encapsulation process [15].

Literature shows that Herbicide oxyfluorfen EC when applied at low dose of 0.1 kg/Ha to rice plants caused phytotoxicity [16], and when applied at 0.2 kg/Ha caused slight phytotoxicity to rice plant six days after transplantation [17–19]. Thus, it may be envisaged that microencapsulation of oxyfluorfen can reduce the toxicity to a greater extent.

Nowadays, interfacial polymerization is used to microencapsulate wide range of active agents within various polymeric shells, like polyureas moreover, polyureas are cheap and safe for environment [20].

No work has been reported on the use of green solvent, N,N-Dimethyldecanamide as Solvent for microencapsulation process and also no work has been reported on the use of various amines for preparing oxyfluorfen microcapsules. N,N-dimethyldecanamide is considered to be green solvent [21].

Objective of Research work was to develop a Microencapsulated formulation of oxyfluorfen that could minimise the phytotoxicity effect. In this work, we studied the formation of core-shell novel polyurea microcapsules containing Herbicide oxyfluorfen via interfacial polymerization of TDI and polydiamines using N,N-dimethyldecanamide as Solvent for the microencapsulation process. The study was conducted by varying core to shell ratio, to study the effect on release dynamics and also on encapsulation efficiency.

We have tried to prepare polyurea microcapsules containing herbicide (oxyfluorfen) by interfacial polycondensation reaction using TDI as first reactant, PVA as emulsifying agent and EDA, HMDA and DETA as the curing agent. The structure of oxyfluorfen and N,N-dimethyldecanamide (solvent) and the amines are represented in *Figure S1*.

## Experimental

### Materials

Oxyfluorfen (98% purity) used as core material was obtained from Willowood chemicals. Toluene-2,4-diisocyanate(TDI) was obtained from SD fine chemicals. Ethylene diamine (EDA) was purchased from Huntsman. Hexamethylenediamine (HMDA) and Diethylenetriamine (DETA) were purchased from Sigma Aldrich. Polyvinyl alcohol having molecular weight range of 13000 to 23000 Da and hydrolysis value of 88 was purchased from Sekisui. N, N-Dimethyldecanamide was purchased from Solvay.

### Preparation of polymer microcapsules

The polyurea microcapsules of oxyfluorfen have been synthesized via interfacial polycondensation process wherein toluene diisocyanate was added to a hydrophobic core in presence of solvent, to form the hydrophobic liquid phase. This phase was then added to an aqueous continuous phase having a colloidal stabilizer, under high shear and emulsified to form an emulsion of liquid droplets of desired particle size suspended in the aqueous phase. To form a polyurea shell around each droplet, diamine or polymaines in the aqueous phase was added to the droplet suspension, thereby forming a suspension of microcapsules.

**Figure 1.**
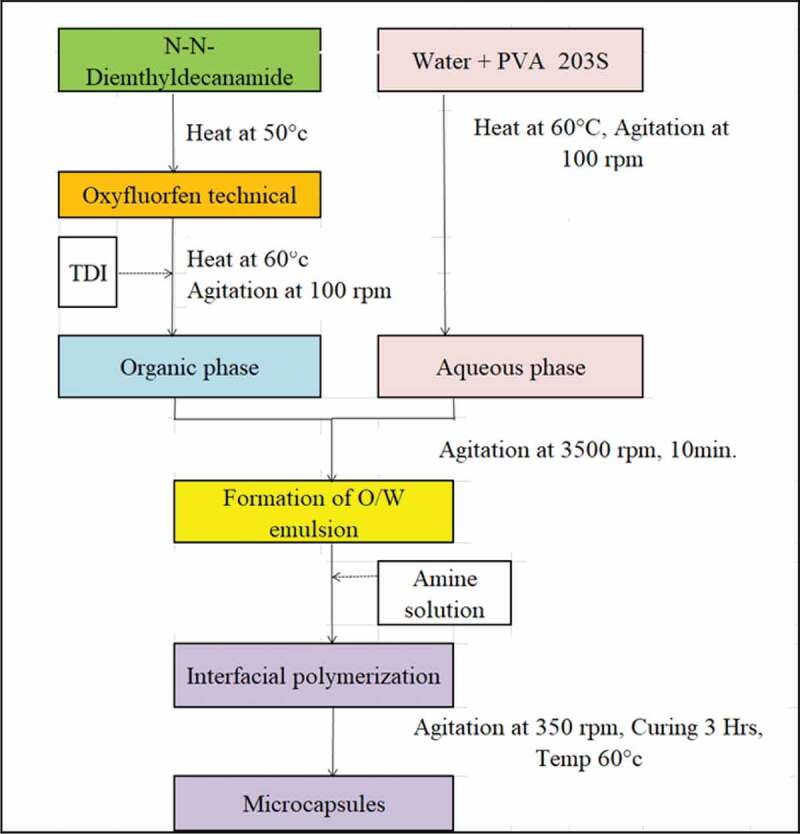
Process flow diagram for preparing microcapsules

The PU capsules were prepared via interfacial polymerisation with TDI monomer and polyamines using N-N-dimethyldecanamide as solvent in an O/W emulsion as shown in Figure 1. The formation of microcapsules was optimized by various experimental parameters like core to shell ratio and are presented in Table 1. TDI was used as the first Reactant while Ethylenediamine, Hexamethylenediamine, Diethylenetriamine subsequently used as second Curing agent.

**Table 1.** Recipes of Microcapsules with various core to shell ratios

Oxyfluorfen (g)	PVA-203S (g)	N-N-dimethyldecanamide (g)	TDI (g)	Amine solution (g)	Water (g)	Core to shell ratio
17	2	10	2.25	1.35 (EDA 60% sol)	67.4	6.5:1
17	2	10	3	1.8 (EDA 60% sol)	66.2	5:1
17	2	10	3.75	2.25 (EDA 60% sol)	65	4:1
17	2	10	1.75	3 (HMDA 40% sol)	66.25	6.5:1
17	2	10	2.3	4 (HMDA 40% sol)	64.7	5:1
17	2	10	2.9	5 (HMDA 40% sol)	63.1	4:1
17	2	10	2	2.5 (DETA 50% sol)	66.5	6.5:1
17	2	10	2.65	3.3 (DETA 50% sol)	65.05	5:1
17	2	10	3.3	4.1 (DETA 50% sol)	63.6	4:1

Preparation of the microcapsules: Example no 1, briefly, 10 gm of N,N-dimethyldecanamide was taken in a 50 ml three-necked round bottom flask and heated to 50°C in a water bath with constant stirring. Seventeen grams of oxyfluorfen technical (98% purity) was slowly poured into it with constant stirring. Heating was continued till oxyfluorfen gets dissolved in the solvent. About 2.25 m of TDI was added slowly at 100 rpm to form organic oil phase and temperature raised to 60°C.

67.4 gms of water was taken in a 250 ml beaker and heated to 60°C. Two grams of polyvinyl alcohol powder was slowly dissolved in it under stirring speed of 100 rpm to form the emulsifier aqueous solution. The organic phase was added to the aqueous phase at 60°C and stirring was continued using rotor/stator homogeniser at 3500 rpm for 10 minutes to obtain an oil in water emulsion. The emulsified sample was then transferred to a 200 ml round bottom flask kept in water bath. About 1.35 g of EDA solution of 60% concentration was slowly added to the reaction mixture in 10 minutes under stirring at 350 rpm and the reaction was continued for 3 hrs at 60°C to obtain microcapsules in slurry form. Slurry suspended in water was directly used for phytotoxicity study.

All the experiments reported here were prepared by the above methodology. Different core to shell ratio (6.5:1, 5:1 and 4:1) were selected for the study. The amount of TDI and EDA were varied to obtain the microcapsules. The reactions were also studied using HMDA (Hexamethylene diamine) and DETA (Diethylenetriamine). Release kinetics and encapsulation efficiencies were evaluated along with safety study on paddy crop.

## Characterization of microcapsules

### FT-IR analysis of microcapsules

The microcapsules suspension was centrifuged to obtain the yellow Capsule powder, and washed first with 30% ethanol aqueous solution, finally the microcapsules were washed thrice with deionized water and dried at room temperature for 24 hrs and used for analysis. FT-IR, recorded on a FT-IR. Bruker model Alpha-2, was used. Oxyfluorfen was directly used. Spectra were recorded between 4000 and 500 cm^−1^ at a resolution of 4 cm^−1^. The functional groups of polyurea microcapsules were examined.

### Measurement of encapsulation efficiency

The encapsulation efficiency of microcapsules was evaluated by mixing a known amount of the reaction mixture with water at room temperature. To this mixture, 50 ml n-Hexane was added for extracting the active ingredient. The amount of the active content in the hexane layer was determined by GLC using DB-1 column (30 m, ID 0.32 µm, Film thickness 0.25 µm) having column temp 225°C, injector and detector temperature of 290°C. Nitrogen Flow rate was 1.5 ml/Min with Split ratio: 20.0. Internal standard used was Dicyclohexyl phthalate. No interference seen of any components using this analysis system. The obtained active content (oxyfluorfen) represents the readily extractable active ingredient (REA) and encapsulation efficiency is expressed as 100-%REA [22].

### Release kinetics study

The release rate of oxyfluorfen from the microcapsules was evaluated from the calibration graph (*Figure S2*). Various known concentrations of oxyfluorfen solutions were prepared in hexane and absorbance (λ_max_) were recorded in the range of 200–600 nm by using UV–Visible spectrophotometer SHIMADZU UV-1601. In this study, a prominent peak at λ_max_ 265 nm was observed. From calibration curve, the unknown concentration of oxyfluorfen was determined. The slope and intercept values were calculated to be 0.0203 and 0.0012, respectively, with R^2^ = 0.9964, using the equation:

y=mx+c

Briefly, 1 gm of microcapsules was transferred to a 250 ml conical flask containing 200 ml of water and the solution was agitated at 150 rpm in an incubator shaker. An aliquot sample of known volume (10 ml) was withdrawn at specific time intervals and added in 50 ml of hexane to extract oxyfluorfen active. The absorbance of hexane layer solution was analyzed by UV-Visible Spectrophotometer at 265 nm. From the absorbance value the concentration of oxyfluorfen was determined using the slope and intercept values. Each aliquot withdrawn was replaced by 10 ml of dilution medium to maintain a constant volume in the flask. All the release experiments were performed in triplicates (*Table S1*).

### Morphological characterization by optical microscope and scanning electron microscopy

The polyurea microcapsules formed were observed initially through optical microscope (Nikon Model Eclipse E200) at 10X resolution for primary confirmation of shell formation. A scanning electron microscope (JEOL JSM IT-200) was used to observe the morphologies of the polyurea microcapsules. Dried microcapsules were mounted on sample holder and vacuum coated using a smart coater for thin gold coating and then taken for SEM analysis.

### Thermogravimetric analysis

Thermal stability of the capsules was studied using TGA (TA Instrument SDT Q600). Thermal stability of the microcapsules was carried out by taking ~7 mg sample in the heating pan and subjected to heating in the range of 24 to 450° C in a flow of 60 ml/min nitrogen. A heating rate of 10°C/min with nitrogen as the purging and protective gas was maintained.

### Particle size measurement

The particle size and size distribution of the polyurea microcapsules were measured using a laser particle size analyser Malvern size model Mastersizer 2000 and the analysis of the samples was done at ambient temperature. The values of volume-based mean diameter were calculated automatically by the instrument. SPAN is calculated by the following equation (1)

(1) $$SPAN=\frac{D_{90-}D_{\text{\textbackslash{}user}10}}{D_{50}}$$

where D90, D10, D50 represent the diameter when the cumulative volume fraction of the measured particles is 90%, 10% and 50%, respectively.

### Field experiments (phytotoxicity assessment)

The prepared polyurea microcapsules (CS) were applied on paddy crop to evaluate the phytotoxicity symptoms. A commercial formulation of Oxyfluorfen 23.5% EC was also applied for comparison. The suitability of microencapsulated product on the plant as control agent was assessed by phytotoxicity assay. The visual phytotoxicity symptoms were evaluated after 10 days of application and the symptoms were rated based on necrosis and Tip burning.

All the phytotoxicity trials were conducted in one plot. Twenty-five-day nursery seedlings were transplanted in the main field. After the third day of transplantation, both the encapsulated product and commercial sample of oxyfluorfen 23.5% EC (Emulsifiable concentrate) were applied by hand-operated knapsack sprayer with flat fan nozzle.

Three sample of Synthesised oxyfluorfen microcapsules labelled as H1, E1, D1 were applied in the field at three different doses along with three doses of commercial sample of oxyfluorfen 23.5% EC after mixing in water as presented in Table 2.

**Table 2.** Treatment details (Core shell ratio 5:1 sample selected)

Treatment no	Product	Does ml/ha
T1	D1 = DETA:TDI Polyurea microcapsules	650 ml
T2	D1 = DETA:TDI Polyurea microcapsules	750 ml
T3	D1 = DETA:TDI Polyurea microcapsules	1000 ml
T4	E1 = EDA:TDI Polyurea microcapsules	650 ml
T5	E1 = EDA:TDI Polyurea microcapsules	750 ml
T6	E1 = EDA:TDI Polyurea microcapsules	1000 ml
T7	H1 = HMDA:TDI Polyureamicrocapsules	650 ml
T8	H1 = HMDA:TDI Polyureamicrocapsules	750 ml
T9	H1 = HMDA:TDI Polyureamicrocapsules	1000 ml
T10	Oxyfluorfen 23.5 EC (market standard)	650 ml
T11	Oxyfluorfen 23.5 EC (market standard)	750 ml
T12	Oxyfluorfen 23.5 EC (market standard)	1000 ml
T13	Untreated Control	

## Results and discussion

### FTIR analysis

*Figure S3* shows the FTIR spectra of polyurea capsules formed using TDI and EDA, blank polyurea microcapsules, core material (oxyfluorfen technical).

To ensure the successful encapsulation of oxyfluorfen, the FTIR spectra of blank polyurea microcapsules (without core) *Figure S3a*, Polyurea loaded oxyfluorfen capsules *Figure S3b*, and core material (oxyfluorfen technical were recorded) *Figure S3c*. Toluene-2,4-diisocyanate reacts with ethylene diamine at the oil-water interface to form an urea linkage. The spectra showed strong hydrogen-bonded N-H stretching vibration at 3300 cm^−1^ in the product and polyurea blank capsules while the vibrations were not seen in oxyfluorfen. The C-H stretching vibrations were observed at 2926 and 2856 cm^−1^. It can be predicted that the reaction between the diisocyanate and the diamine was complete because the IR spectrum did not exhibit the NCO peak at 2270 cm^−1^ while, a new peak of N-H and C = O absorption bands appeared, indicating an interaction between TDI and EDA. Polyurea capsules also exhibited strong carbonyl stretching at 1650 cm^−1^. Core material, *Figure S3c*, exhibited N-O stretching vibrations at 1576 cm^−1^. Absence of this peak in polyurea blank capsules *Figure S3a* and appearance in the product *Figure S3b* further indicates that the polyurea capsules are formed containing the core material oxyfluorfen.

### Encapsulation efficiency

The oxyfluorfen microcapsules were prepared by interfacial polymerization. The reaction takes place at oil-water interface and formed Polyurea oxyfluorfen capsules. The core to shell ratio and type of various shell-forming material were studied. Among the amines tested the encapsulation efficiency was found to be highest with Ethylenediamine, (97.47%–98.2%), followed by DETA (96.72%–97.41%) and HMDA (92.6%–94.6%). HMDA formed polyurea capsules had lowest encapsulation efficiency compared to EDA and DETA polyurea capsules. This can be explained by the fact that as the methylene groups of the amines used in the reaction is increased, reaction is taking place slowly resulting in lower encapsulation of the product.

**Figure 2.**
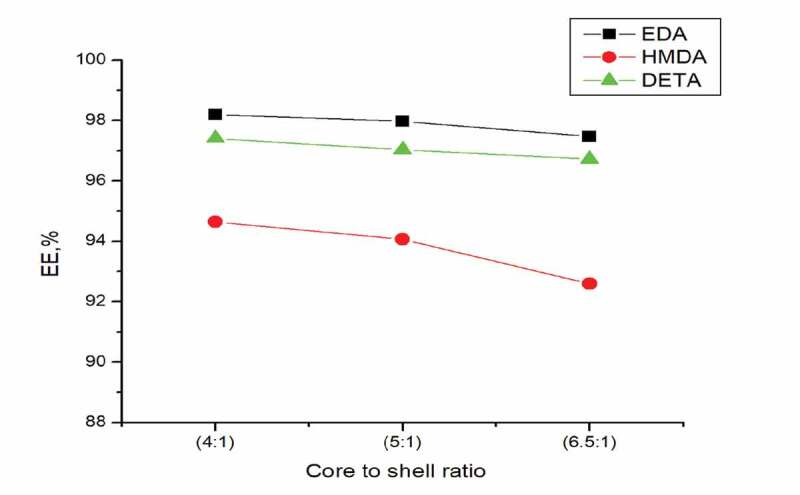
Effect of Encapsulation efficiency using amines and core to shell ratio

Another factor that affected the encapsulation efficiency is the core to shell ratio. Figure 2 demonstrated the effect of core to shell ratio on the encapsulation efficiency. The core to shell ratio used were 6.5:1, 5:1, and 4:1. The encapsulation efficiency was found to decrease with increasing core to shell ratio. This may to be due to the fact that the amount of Oxyfluorfen was exceeding and remains unencapsulated during the reaction or can be explained that with reduction in quantity of wall material the resultant polyurea membrane would not completely cover the core material. It is more visible with HMDA polyurea capsules.

The results show that encapsulation of oxyfluorfen is based on hydrophobic interaction between oxyfluorfen and the hydrophobic segments of toluene diisocyanate and hydrophilic segments of cross-linking amine. The hydrophobic interactions are strong enough to remain in water or in ionic media. It is also known that individual hydrophobic or hydrophilic monomer cannot form particles with core material under the same experimental conditions. Thus, it can be said that the capsules have oxyfluorfen as the core and polyurea as the shell formed by reaction of hydrophobic monomer (TDI) with hydrophilic monomer, i.e., cross-linking amine.

### Release kinetics of oxyfluorfen in water

**Figure 3.**
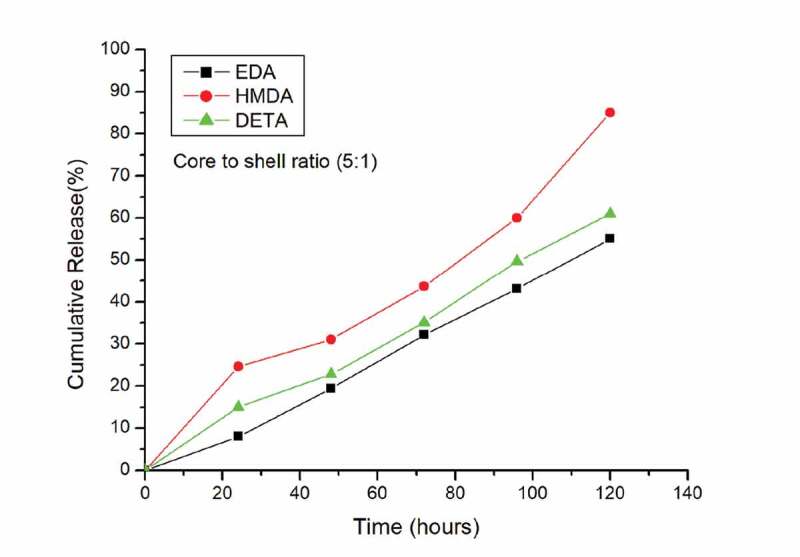
Release behaviour of different PU capsules having core to shell ratio of 5:1

Release study of oxyfluorfen from the microcapsules was evaluated for a period of 120 hrs The release studies were done in triplicates. *Figure S4*, Figure 3, *Figure S5* represents the release behaviour of the oxyfluorfen polyurea microcapsules in the dissolution medium, i.e., water. The microencapsulation was carried out by varying core to shell ratio and also using different amines to investigate the effect of on the release rate of the oxyfluorfen.

It can be clearly observed that all the microcapsules released Oxyfluorfen rapidly in the initial stage from 7% to 34%. The microsphere formulations exhibited burst release at the beginning of the release experiments. This is due to the core material which remains unencapsualated during the reaction and secondly the huge gap of concentration inside and outside the wall membrane would trigger the rapid release at the initial stage [23]. In case of HMDA prepared polyurea capsules the initial Release was very fast and the final release amount reached to greater than 90% within 120 hrs for core to shell ratio of 6.5:1. *Figure S4.*

Comparison of all the core to shell ratio, i.e. (6.5:1,5:1,4:1), *Figure S4*, Figure 3, *Figure S5*, it is seen that in all the core to shell ratios, Faster release was seen with polyurea capsules prepared using HMDA as curing agent. Fast release in HMDA: diisocyanate polyureacapsules can be attributed to the larger alkyl chain length of HMDA compared to DETA and EDA as shown in *Figure S1* and also to surface morphology. From the results it is concluded that microcapsules with thicker walls are formed using EDA and DETA, compared to HMDA polyurea capsules, which explains the high release of core material from HMDA polyurea capsules.

In addition, it is observed that core to shell ratio had great influence on the release of oxyfluorfen. Fast release was observed at core to shell ratio of 6.5:1, Maximum release content of oxyfluorfen was found to be 94.4% for the core to shell ratio of 6.5:1 (HMDA-based polyurea capsules) *Figure S4* which produces the thinner shell material which results in the high amount of diffusion of the Oxyflurofen through shell as compared to other core to shell ratios.

Slow release was observed at core to shell ratio of 4:1. *Figure S5*. While minimum release content of oxyfluorfen was found to be 48% for core to shell ratio of 4:1 (EDA-based polyurea capsules) which can be attributed to increased wall thickness of the capsules. This behaviour may be explained by the different characteristics of the oxyfluorfen microcapsules as a result of different core to shell ratio and also on the type of curing agent used for microencapsulation process. Sustained release is seen in polyurea capsules prepared using Ethylenediamine as the curing agent and release is more consistent at core to shell ratio of 5:1 as observed in Figure 3.

So, changing the core to shell ratio is an effective way to precisely control the release of the core material.

## Study of surface morphology

### Optical microscope

**Figure 4.**
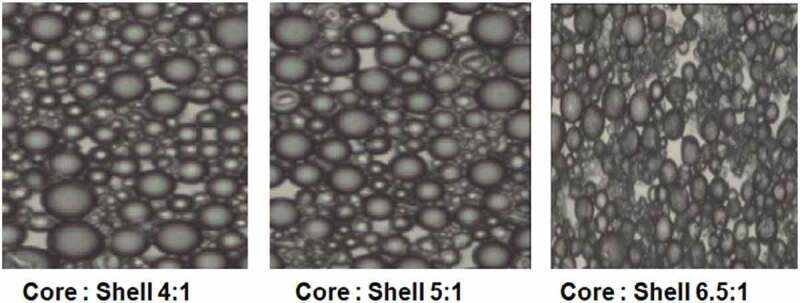
Optical images of EDA polyurea capsules

The surface morphology of oxyfluorfen PU microcapsules was observed under optical microscope at 10× resolution for primary confirmation of shell formation at different core to shell ratio Figure 4, represents a typical optical microscopic image of polyurea microcapsules formed during reaction of EDA and Toluene diisocyantate in presence of solvent N-N-dimethyldecanamide. Images indicate that the reaction has taken place between EDA/TDI and polyurea capsules are formed. As the core to shell ratio is increased thin wall formation is observed and this also explains the different release behaviour of core from the shell.

### Scanning electron microscope (SEM)

**Figure 5.**
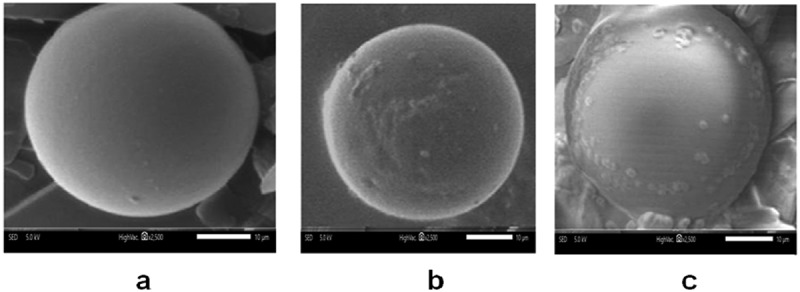
SEM images of (a) DETA PU, (b) EDA PU and (c) HMDA PU

Scanning electron microscope (SEM) images of oxyfluorfen PU microcapsules are shown in Figure 5. For core shell ratio of 5:1, observing the morphology of microcapsules has great significance to the research of micro-encapsulation [24].

Figure 5(a) shows the prepared polyurea microcapsules with Diethylenetriamine. The microcapsules are spherical with compact outer surface. Figure 5(b) shows polyurea microcapsules with ethylenediamine. The microcapsule was spherical and had rough surfaces in comparison with other samples. Figure 5(c) shows HMDA (Hexamethylenediamine) based microcapsules which were found to be somewhat spherical, wrinkled and had obvious depressions.

The shrinkering of the microcapsules may be due to reduction in internal area caused by release of solvent and core material. Wrinkling may be due to the interaction of inhomogeneous reaction kinetics, fluid-induced shear forces, and shell-determined elastic forces [25]. SEM micrographs also showed the presence of crystals in some preparations. It is suspected that these crystals are derived from unencapsulated Oxyfluorfen. GLC analysis indicated that small amount of oxyfluorfen ranging from 2.03% to 7.4%.

The surface morphology of HMDA-prepared microcapsules is envisaged to have different oxyfluorfen release behaviour compared to DETA and EDA-based microcapsules

### Thermo gravimetric analysis

*Figure S6* displays the TGA diagrams of core material and polyurea microcapsules synthesized from various amines. Weight loss of each sample also verifies the formation of polyurea microcapsules with high thermal stability [26,27]. The initial weight loss of 36%-49% in the range of 110°C–232°C is attributed to the volatilization and degradation of the core material oxyfluorfen. Second weight loss of about 51%-55% in the range of 232°C–350°C can be attributed to the decomposition of polyurea wall material. The mass loss of oxyfluorfen begins at 180°C and ends at 280°C corresponding to the volatilization of oxyfluorfen.

### Particle size measurement

**Figure 6.**
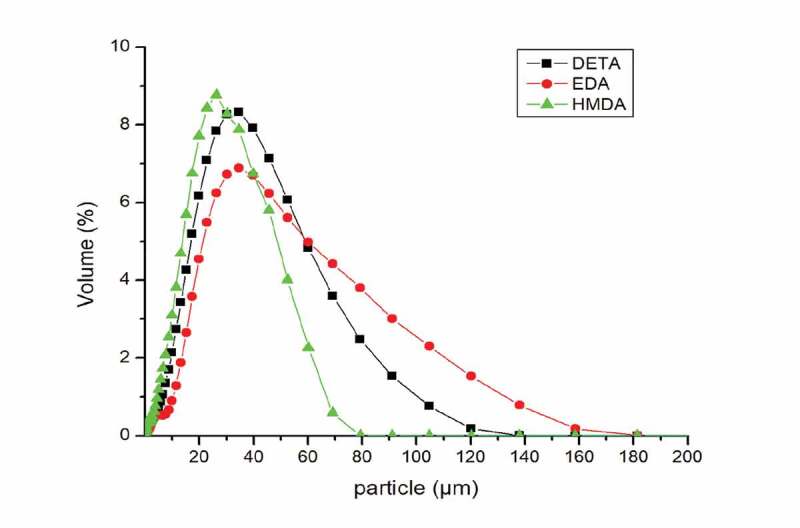
Particle size distribution of PU microcapsules using various diamines

The size distribution and particle size of the prepared polyurea microcapsules using amines EDA, DETA and HMDA having core to shell ratio of (5:1) is shown in Figure 6.

Microcapsules size is important parameter that governs the release rate of core material. It is clearly observed that all the microcapsules were in the range of 20-50 µm. The curve profile presented a better normal distribution. EDA-based capsules had mean diameter of 37.495 µm with span of 1.735. DETA-based capsules had mean diameter of 30.769 µm with span of 1.824 and HMDA-based capsules had mean diameter of 28.28 µm with span of 3.079.

It has been observed that polyurea capsules prepared from HMDA had narrow distribution range. Polyurea capsules prepared from EDA had a broad distribution range. This can be attributed due to the fact that as the methylene group is increased the size distribution becomes narrow which may be due to fact that addition of EDA resulted in thicker microcapsules due to its superior reactivity than HMDA and DETA [28].

### Phytotoxicity assessment

**Figure 7.**
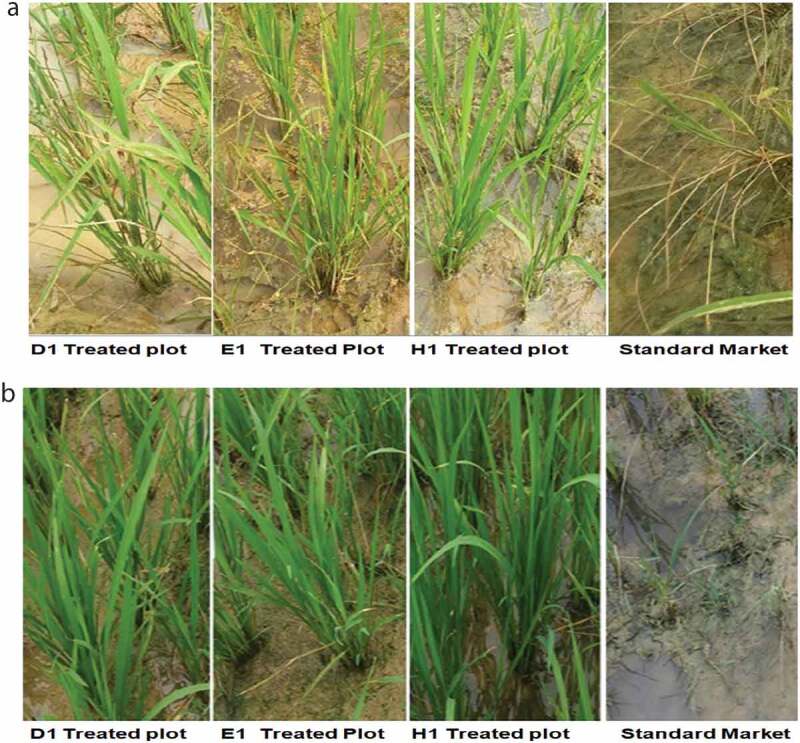
Phytotoxicity assessment after 10^th^ day of treatment

The phytotoxicity assessment was made based on rating scale as presented in *Table S2* [29]. When polyurea microcapsules (CS) sample of oxyfluorfen was applied in the field along with commercial sample of oxyfluorfen 23.5% EC, initial yellowing and tip burning was observed after second day of application, but in all the treatments of prepared microcapsules, fast recovery was observed when compared with commercial sample as seen in Figure 7(a). Recovery was faster in EDA-based polyurea capsules Followed by DETA-based polyurea capsules and HMDA-based polyurea capsules indicating that type of monomer has a significant role in the release of core from the shell. The marketed standard sample of oxyfluorfen EC completely damaged the paddy crop after third day at high dose level of 650 ml/ha (1.3 ml in 1000 ml water), 750 ml/ha (1.5 ml in 1000 ml water) and at 1000 ml/ha (2 ml in 1000 ml water) as presented in *Table S3 and S4*.

On the 30^th^ day, it was observed that the crop treated with microencapsulated product exhibited improved growth while crop treated with market sample was completely damaged and dried as seen in Figure 7(b).

## Conclusion

In this study, polyurea microcapsules containing oxyfluorfen were prepared by interfacial polymerisation reaction using TDI and amines such as EDA, DETA and HMDA using N-N-Dimethyldecanamide as the solvent for encapsulation process. The study indicated that the Core to shell ratio had impact on release behaviour of the core from the polyurea microcapsules and the release can be controlled by core to shell ratio. It also depended upon the type of amines used, sustained release was observed with EDA-based polyurea capsules. Encapsulation efficiency of the herbicide from the polyurea microcapsules was depended on the type of amines used and also on core to shell ratio. The encapsulation efficiency was found to decrease with increasing core to shell ratio. EDA prepared polyureacapsules had the highest encapsulation efficiency of 98.2%. The SEM study indicated that the surface of the polyurea microcapsules was different when reacted with different types of amines. The phytotoxicity assessment indicated that the polyurea microcapsules were safe to use on paddy crop when compared to the market standard of oxyfluorfen Emulsifiable concentrate. Hence oxyfluorfen microcapsules have the potential to be developed as safe and alternative environment-friendly formulations and help the researchers further to explore this area.

## Supplementary Material

Supplemental Material
