# Supplementary material for: Design and application of polyurea microcapsules containing herbicide (oxyfluorfen)
Source: Des Monomers Polym. 2020 Sep 8;23(1):155–63. doi: 10.1080/15685551.2020.1816344 (PMC7738284; doi:10.1080/15685551.2020.1816344)
Supplement: Supplemental Material [file TDMP_A_1816344_SM2114.pdf]

Supporting Information

Design and application of polyurea microcapsules containing herbicide (oxyfluorfen)

Jayprakash Rao[1,2], Amar Nath Chandrani[1], Anil Powar[1], Sudeshna Chandra[2*]

[1]*Indofil Industries limited, Off Swami Vivekananda Raod, Azad Nagar, Sandoz Baug,Thane-400607 India*

[2]*Department of Chemistry, Sunandan Divatia School of Science, SVKM's NMIMS (Deemed to be) University, V. L. Mehta Road, Vile Parle (West), Mumbai, 400056 India*

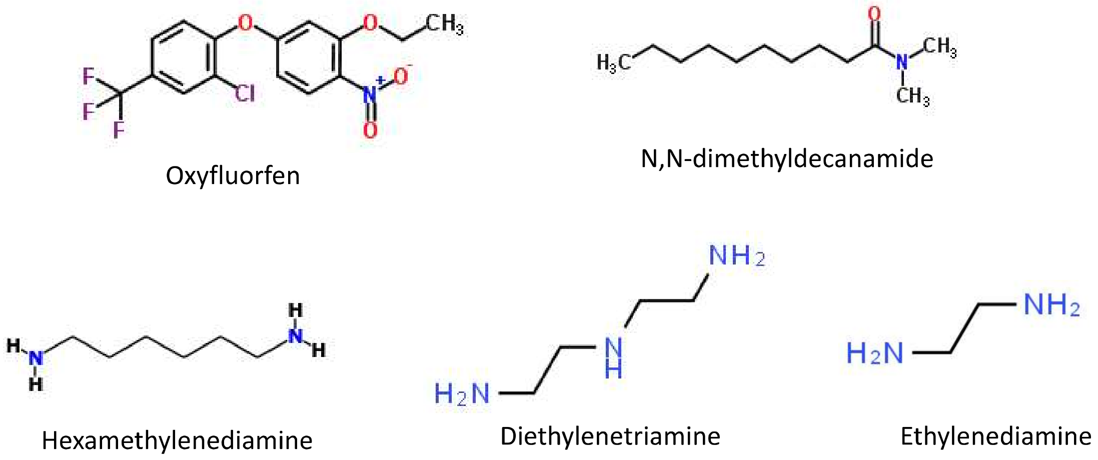

Figure S1: Chemical structures of various components

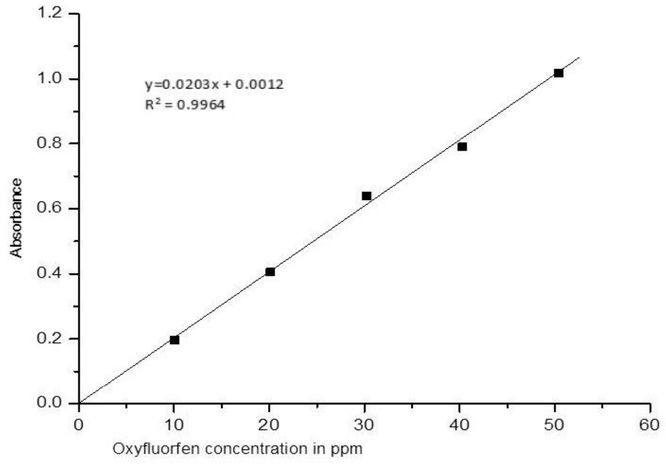

Figure S2: Calibration curve of Oxyfluorfen

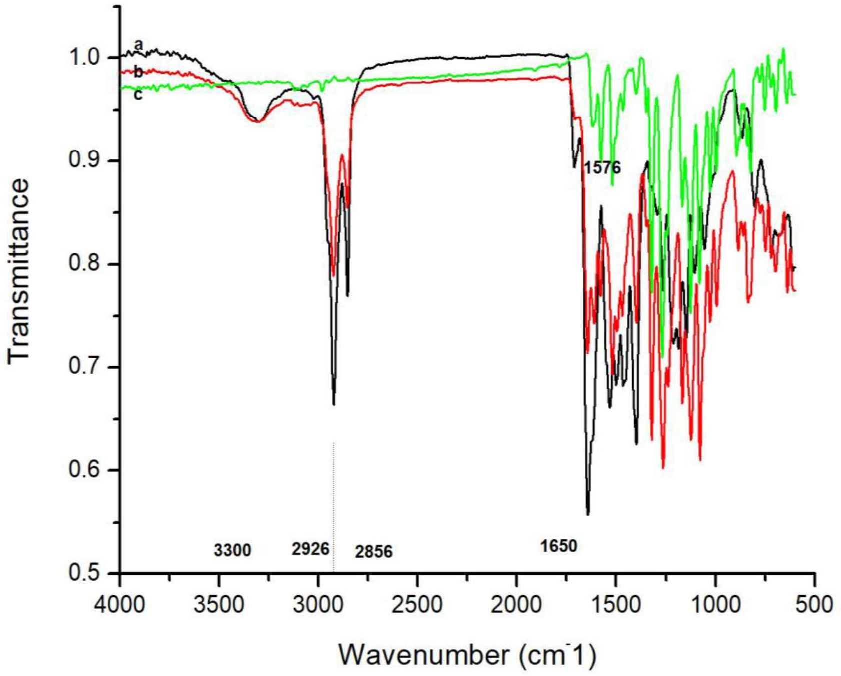

Figure S3: FTIR spectra of (a) Blank Polyureacapsules, (b) Product, and (c) Core material

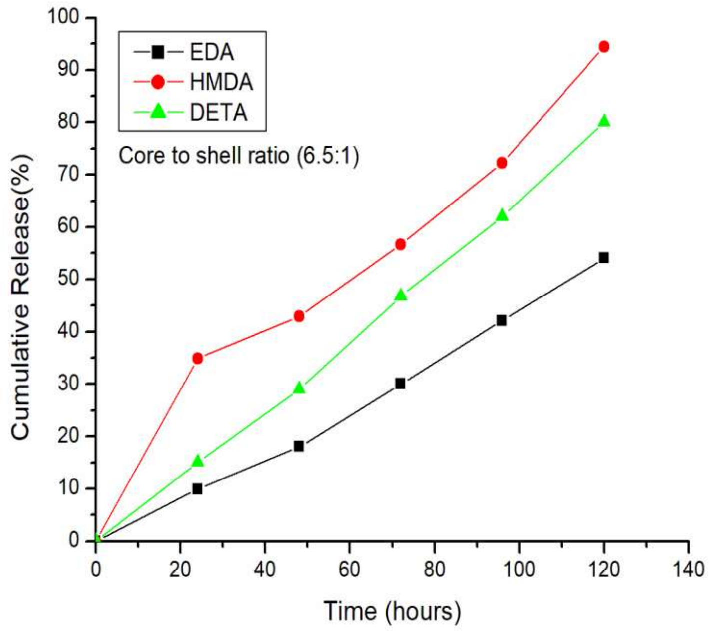

Figure *S4. Release behaviour of different PU capsules having core to shell ratio 6.5:1*

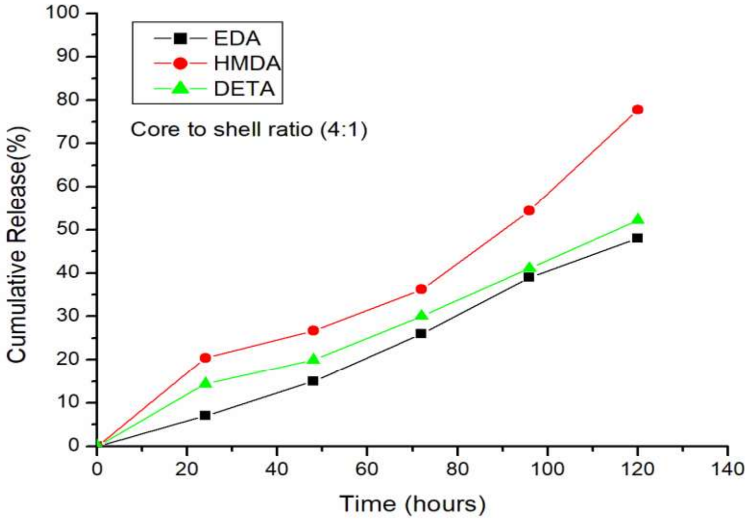

Figure S5. Release behavior of different PU capsules having core to shell ratio of 4:1

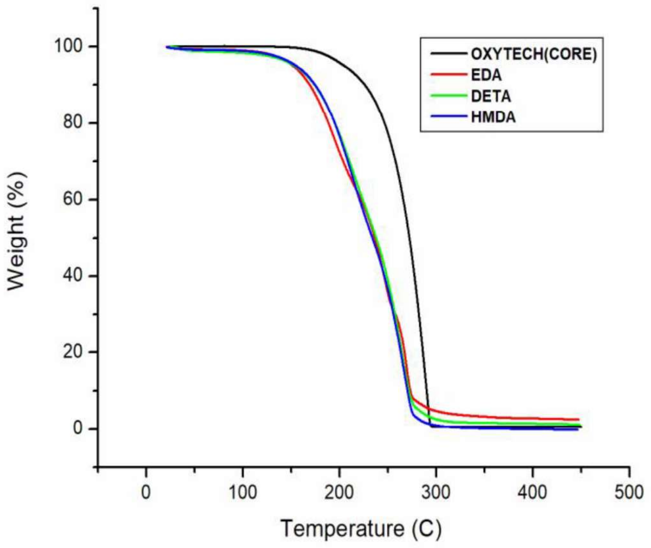

Figure S6. TGA thermograms of core material and PU microcapsules prepared from EDA, DETA and HMDA (core to shell ratio 5:1)

Table S1 : Release behaviour of different PU microcapsules having varying core to shell ratio

Curing agent	Core to shell ratio	Release kinetics study at various time intervals									
		24 hrs		48 hrs		72 hrs		96 hrs		120 hrs	
		Mean	SD (±)	Mean	SD (±)	Mean	SD (±)	Mean	SD (±)	Mean	SD (±)
HMDA	6.5:1	34.9	0.779	42.9	0.589	56.7	0.374	72.3	0.624	94.4	0.419
DETA	6.5:1	15.03	0.356	29	0.712	46.74	0.74	62	0.408	80	0.45
EDA	6.5:1	9.9	0.214	18	0.327	30	0.432	42.1	0.327	54	0.374
HMDA	5:1	24.6	0.864	31	0.707	43.6	0.668	60	0.816	85	0.535
DETA	5:1	14.97	0.616	22.8	0.698	35	0.497	49.61	0.86	60.97	0.694
EDA	5:1	8	0.141	19.4	0.327	32.1	0.356	43.1	0.455	55	0.408
HMDA	4:1	20.4	0.424	26.7	0.249	36.2	0.589	54.4	0.589	77.8	0.356
DETA	4:1	14.36	0.909	19.9	0.535	30	0.356	39.9	0.638	52.21	0.883
EDA	4:1	7	0.374	15	0.432	26	0.424	39	0.497	48	0.408

S.D = Standard deviation, where n=3

Table S2: Phytotoxicity assessment of the paddy crop based on ratings

Rating	Crop Responses/ Crop Injury (%)	Verbal Description
0	0	No symptoms
1	1-10	Very slight discoloration
2	11-20	More severe, but not lasting
3	21-30	Moderate and more lasting
4	31-40	Medium and lasting
5	41-50	Moderately heavy
6	51-60	Heavy
7	61-70	Very heavy
8	71-80	Nearly destroyed
9	81-90	Destroyed
10	91-100	Completely destroyed

Table S3: Phytotoxicity assessment (Necrosis)

**Treatment no**	**Product**	**Dose/ ha ml**	***NECROSIS / BURNING*** **Days after application**								
			**0**	**1**	**3**	**5**	**7**	**9**	**11**	**15**	**30**
T1	D1	650	0	3	4	4	3	0	0	0	0
T2	D1	750	0	3	5	5	3	0	0	0	0
T3	D1	1000	0	3	5	5	3	0	0	0	0
T4	E1	650	0	2	3	5	3	0	0	0	0
T5	E1	750	0	2	3	5	3	0	0	0	0
T6	E1	1000	0	2	5	5	4	0	0	0	0
T7	H1	650	0	3	4	3	3	0	0	0	0
T8	H1	750	0	3	4	3	4	0	0	0	0
T9	H1	1000	0	3	5	5	4	0	0	0	0
T10	Oxyfluorfen 23.5 EC	650	0	7	9	9	9	9	9	10	10
T11	Oxyfluorfen 23.5 EC	750	0	7	9	9	9	9	9	10	10
T12	Oxyfluorfen 23.5 EC	1000	0	7	9	9	9	9	9	10	10
T13	Untreated Control	-	0	0	0	0	0	0	0	0	0

Table S4: Phytotoxicity assessment (Tip burning)

Treatment no	Product	Dose/ ha ml	*TIP BURNING* Days after application 0	1	3	5	7	9	11	15	30
T1	D1	650	0	3	5	5	3	0	0	0	0
T2	D1	750	0	3	5	5	3	0	0	0	0
T3	D1	1000	0	5	5	5	3	0	0	0	0
T4	E1	650	0	4	5	5	3	0	0	0	0
T5	E1	750	0	4	5	5	3	0	0	0	0
T6	E1	1000	0	5	5	5	3	0	0	0	0
T7	H1	650	0	4	5	3	2	0	0	0	0
T8	H1	750	0	5	5	4	2	0	0	0	0
T9	H1	1000	0	5	5	4	2	0	0	0	0
T10	Oxyfluorfen 23.5 EC	650	0	5	8	9	9	9	9	10	10
T11	Oxyfluorfen 23.5 EC	750	0	6	8	9	9	9	9	10	10
T12	Oxyfluorfen 23.5 EC	1000	0	6	8	9	9	9	9	10	10
T13	Untreated Control	-	0	0	0	0	0	0	0	0	0
